# Eclampsia Infantum

**Published:** 1873-05

**Authors:** T. Curtis Smith

**Affiliations:** Middleport, Ohio


### ECLAMPSIA INFANTUM.

BY T. CURTIS SMITH, M.D., MIDDLEPORT, OHIO.

Eclampsia infantum is recognized as a disease affecting infants, and children at any age prior to puberty, but as occurring most frequently within the first half of the period of infancy. It is an affection that quickly spreads dismay through the family circle; the physician is hastily summoned, and he, too, may partake of the alarm, unless he can readily assure himself that the danger is only apparent and not real. Indeed, these cases are often critical, and not a few of them terminate fatally. To demonstrate the diagnostic and prognostic features of these cases shall be one of the objects of this paper, in a brief space.

Eclampsia infantum is of different varieties, which may, for all practical purposes, I think, be divided into sympathetic and symptomatic—the former being the result of sympathy with a deranged or diseased organ or tissue in any part of the system exterior to the cerebro-spinal axis; the latter being or constituting a grave symptom in the course of diseases affecting some portion of that axis, structurally.

Meigs and Pepper, in their most excellent work on "Diseases of Children," divide the disease into essential, sympathetic, and symptomatic, with the following explanation, viz.: "The* first two classes are unaccompanied by appreciable lesions of the nervous centres, while the third is called symptomatic, because it includes cases of convulsions, which are the sign or symptom of an appreciable lesion of the cerebro-spinal axis—as, for instance, those which occur in the course of meningitis, tubercular disease, hydrocephalis, apoplexy, &c. In idiopathic or essential convulsions, the cause of the attack acts directly upon the nervous centres, while in those to which the term sympathetic is applied, the cause lies in the influence of or effect upon the brain or spinal marrow, of disease of some other origin; to the latter class belong the convulsions which occur in the course of pneumonia, * * eruptive fevers, &c." To me, it seems that while there is room for such a subdivision, especially with reference to its bearing on the prognosis, that never-

*See page 493, Fourth Edition.

theless, the essential form, as described by Meigs and Pepper, might well be included under the one term *sympathetic;* for what is it, after all, but sympathy of the nervous system with the diseased organ when convulsions result from pneumonia, bronchitis, hepatitis, &c.?

The symptomatic eclampsia should be treated of when treating of diseases affecting the nerve-centres; hence, we shall only speak of the sympathetic form in this paper, and that very briefly.

Sympathetic eclampsia, though not constituting a disease *per se,* results from lesions in, or irritations of, organs or tissues exterior to the cerebro-spinal axis, but which act more or less directly upon that axis through reflex action. It therefore includes all cases of eclampsia in infants and children not produced by direct lesion of a nerve-centre.

The exciting causes producing this form are very numerous and varied, and the prognosis is favorable or unfavorable in proportion to the gravity of the lesion or irritation giving rise through sympathy to the convulsive action. The convulsions are caused by burns, scalds, slight and severe wounds; by violent pain from any cause; by severe cold or heat, especially to the head; the evolutions of dentition, decaying or ulcerating teeth, or irritation by a remaining tooth fang; local inflammations, as from a furuncle, carbuncle, whitlow, or blister produced by therapeutic agents; by anger, fright, long-continued anxiety; by intestinal worms,* or indigestion, acidity of the stomach, impaction of the colon or rectum; by suppressed exanthemata, as of rubeola, scarlatina, or by the tardy appearance of such rashes on the surface; by retention of urine, or a failure of the kidneys, liver or other organ to perform its functions properly, or by the systemic absorption of secretions that should be eliminated; by morbific poisons, as malarial, diphtheritic, &c. Again, they are frequently caused, through sympathy, by organic lesions of any important organ, as of the lungs, bronchia, liver, stomach, kidneys, &c., and that, too, without special regard to whether the organic change be inflammatory, tubercular, or fatty degeneration,

*I know of two cases in one family where it was produced three times in one child, and twice in another, by eating largely of blackberries, the indigestible seeds seeming to be the exciting cause in each instance. It is often caused by eating green fruits.

for the experience of morbid pathology teaches that they may arise from any of these lesions, and especially in proportion to the derangement of the functions of the organ involved.

Many other and varied exciting causes could be enumerated, but certainly we have referred to enough to show the great multiplicity of morbid conditions that may produce this form of eclampsia infantum.

The predisposing causes are not so numerous, and not always readily recognized. Of these, age seems among the most prominent. Dr. James Whitehead, of Manchester, England,* gives a very clear and graphic account of a case of "convulsions in utero," in which the attacks recurred every four or five minutes, and continued to do so for "five hours—from six to eleven P.M." Such cases, of course, must be rare.

As stated before, the child is most liable to eclampsia during the first three years of its age. In my own experience it has occurred most frequently after the first six months, and between that and the thirtieth month of infancy. Meigs and Pepper state that of ninety-one cases† noted by them, nineteen occurred in the first year, twenty-six in the second year, twenty the third and fourth year, twenty-three from the fourth to the ninth year, and three from the ninth to the thirteenth year—thus showing that by far the greater proportion of cases occur in very early life. This may readily be accounted for by the well-known fact that in infancy the development of the nervous system is vastly greater proportionately than the remaining portions of the physical economy, and is more easily impressed and influenced either by natural or morbid processes than in later life.

Dr. West states that within the first year the deaths from convulsions constituted (in the city) 73.3 per cent. of the total mortality from diseases of the nervous system, and from the first to the third year 24.9 per cent., and so on decreasing till it was but eight-tenths of one per cent. after the fifteenth year. He attributes the frequency of eclampsia in children to the great predominance of

*See "Compendium of Medical Science," Part I., for 1868, page 148, taken from the *British Medical Journal* for July 27, 1867, page 59.

†See page 494, Fourth Edition.

the spinal over the cerebral system, and to the imperfect development of the brain.*

Sex seems to make but little, if any, difference in the frequency of attacks as a predisposing cause. Other causes are a delicate nervous organization. A hereditary influence is often observable in some families of high nervous organization. Occasionally we meet with a family in which nearly every child has at some period been the subject of such attacks. Meigs and Pepper mention a family in which five out of six children were the subjects of attack, four of them being repeated attacks. Dr. Robert P. Harris, in vol. xi., page 281, of the *American Journal of Obstetrics,* gives statistics of thirteen families, in which fifty-nine children had been born, forty-two still living at date of his writing. Of the fifty-nine children, thirty-seven were subjects of infantile eclampsia, and five out of the number died while affected with it.

The symptoms I shall pass over without notice, as they are too apparent to require description.

For a diagnosis, as to whether the convulsions are sympathetic or symptomatic, the practitioner must depend largely on the history of the case. If it has a history of disease affecting the cerebro-spinal system, its nature may be readily made out. But when such diseases begin with convulsive action, there is greater difficulty in making a satisfactory diagnosis. Close observation of the symptoms, with a little time, will decide their nature. In a few cases, however, their nature, even when of this type, may be made out at once.

*Prognosis.*—The prognosis of eclampsia is favorable or grave in proportion to the gravity of the exciting cause, or in proportion to the importance of the organ that has excited the convulsions, and not less in proportion to the removability of the exciting cause.

In the form denominated by Meigs and Pepper as idiopathic or essential, the termination is favorable in the vast majority of cases, for in this class of cases the exciting cause is easily removed, it usually being such as intestinal worms, dentition, acidity of the stomach, or some local irritation acting upon the cerebro-spinal system through reflex sympathy.

* Loc. cit.

But in those forms which they denominate sympathetic, and which occur in connection with, or result from, serious visceral or local disease, or from the tardy appearance of a rash or its sudden suppression, or while the system is greatly depressed by diphtheritic or variolus poison, &c., the prognosis is less favorable. Not but that the convulsions may quite readily be kept in abeyance generally, but the additional shock to the nervous system adds to the depression already existing, which is usually great, thus rendering the prospect of recovery less favorable than is the case where no serious lesion is exciting the eclampsia. Thus, in cases of pneumonitis, bronchitis, hepatitis, &c., if eclampsia occur, it may always be looked upon as a grave symptom, especially if it occur after the disease is fully established and the system already seriously depressed by it. I grant that eclampsia may, and frequently does, occur at the inception of such diseases from shock to the nervous system, and the cases go on through the course of the disease without the eclampsia having, so far as we can observe, added but little to the real gravity of the case; but this can hardly be so, according to my experience, if the convulsions occur after the full establishment of such morbid process.

*Treatment.*—On the treatment I shall be very brief. The indications are to first overcome the spasm; second, to give some agent or agents that will act as a prompt nervous sedative; while at the same time, the third and most important, means are being used to remove, if possible, the exciting cause of the eclampsia.

The first indication the physician is seldom present to meet, viz.: that of breaking up the convulsive action. To accomplish this, however, cold water may be dashed in the face, or dashed upon the course of the spine; the feet put alternately into warm and cold water in quick succession and frequently repeated; water alternately warm and cold may be also applied over the breast and stomach; a light draft of ammonia may be applied to the nose; in fact, any means may be used that will cause a sudden counter-shock to the system. Should the attack continue long, anæsthetics may be used with advantage. In one case in which the convulsion continued persistently for seven hours, after using repeatedly all the usual means, I gave the child, aged two years, morphia sulph., grs. one-eighth, every thirty minutes for three doses, which entirely controlled the attack. A few months later, I repeated the same process, with a like result, in the same child. Usually, I should fear to use morphia at all in eclampsia infantum, except where pain was the cause, and I confess I did in this case with some misgiving; but all other agents had utterly failed, and as a dernier resort (as it seemed to me) I gave the morphia. That was before brom. potass. came into such general use, and I did not use it in this case at any time. In another case where the convulsion persisted for thirty minutes, I applied a towel out of ice-water to the entire spine, with the immediate effect of causing the convulsion to cease.

The second and third indications I have usually met at the same time: the former by the free administration of bromide of potassium, always alone, unless there is evidence that the convulsion has been caused by severe pain or shock, in which case from the 1-32 to the $\frac{1}{8}$ of a grain of morphia sulph. may be given with advantage—and this sometimes needs to be combined with a stimulant, especially if the shock is very great. But to meet the second indication, I know of nothing to compare in value, in a vast majority of cases, with bromide potassium, given in doses sufficient to influence the nervous system promptly—say five to thirty grains every twenty minutes to a child one year old, and more proportionately in older infants.

The third indication in all these cases may be attended to at the same time, viz.: the removal of the cause. This may be done by first searching carefully for the cause, and then applying the necessary remedy. If it be in the mouth, lance the gums or remove the offending tooth. If an abscess, open it freely. If acidity, give an antacid purgative; and if from undigested food, evacuate the stomach quickly and move the bowels by the prompt use of an enema of suds or weak salt-water. If worms, evacuate the bowels by enema of suds and a ʒi. or ʒii. of turpentine; give a prompt anthelmintic, and wet a flannel or other cloth with turpentine and tack it on the outside of the clothing at the upper part of the breast, so the child may breathe the inhalations of it. This will prevent the worms from rising in the child's throat while other agents are having time to act. Meantime, in every case open well up all the secretions of the different organs, and don't neglect all the while the free use of brom. potass.

If malaria is the cause, use quinia freely. In fact, seek the exciting cause, and remove it promptly.

If they result from pneumonia, bronchitis, eruptive fevers, &c., control the convulsions with nervous sedatives or nervous stimulants as they may require, and then treat the real disease the same as though no eclampsia had ever occurred, except that the nervous system should be carefully watched, and any indications of eclampsia promptly met, and, if possible, its recurrence prevented.

